# Piecing Together Phenotypes of Brain Injury and Dysfunction in Obstructive Sleep Apnea

**DOI:** 10.3389/fneur.2012.00139

**Published:** 2012-10-09

**Authors:** Sigrid C. Veasey

**Affiliations:** ^1^Center for Sleep and Circadian Neurobiology, Department of Medicine, University of Pennsylvania School of MedicinePhiladelphia, PA, USA

**Keywords:** cognition, cytokines, endoplasmic reticulum, hypoxia, memory, NADPH oxidase

## Abstract

Obstructive sleep apnea (OSA) is a highly prevalent condition that is associated with significant neurobehavioral impairments. Cognitive abnormalities identified in individuals with OSA include impaired verbal memory, planning, reasoning, vigilance, and mood. Therapy for OSA improves some but not all neurobehavioral outcomes, supporting a direct role for OSA in brain dysfunction and raising the question of irreversible injury from OSA. Recent clinical studies have refined the neurobehavioral, brain imaging, and electrophysiological characteristics of OSA, highlighting findings shared with aging and some unique to OSA. This review summarizes the cognitive, brain metabolic and structural, and peripheral nerve conduction changes observed in OSA that collectively provide a distinct phenotype of OSA brain injury and dysfunction. Findings in animal models of OSA provide insight into molecular mechanisms underlying OSA neuronal injury that can be related back to human neural injury and dysfunction. A comprehensive phenotype of brain function and injury in OSA is essential for advancing diagnosis, prevention, and treatment of this common disorder.

## Obstructive Sleep Apnea: Clinical Overview

Obstructive sleep apnea (OSA) is a chronic sleep state-dependent breathing disorder. This is a prevalent condition, observed in 5–7% of general (adult and children) populations within most developed and developing countries (Young et al., 1993; Punjabi, 2008). The predisposing factor for OSA is a highly collapsible upper airway, and the pathophysiology of the disorder may be summarized as intermittent occlusions of the upper airway occurring exclusively in sleep. In wakefulness upper airway patency in individuals with OSA is maintained by upper airway dilator muscle activation. A normal physiological effect of sleep is reduced tone in many muscle groups, including upper airway dilator muscles (Remmers et al., 1978). Upon arousal, pharyngeal patency is restored (Remmers et al., 1978; Dempsey et al., 2010). These obstructive sleep-disordered breathing events occur 5–120 times/h of sleep in patients depending upon severity of OSA. Because of muscle atonia and reduced chemosensitivity in rapid-eye-movement (REM) sleep, the frequency of apneas and the severity of oxygen desaturations are frequently more severe in REM sleep (Skatrud et al., 1990).

Overall, most conditions that predispose an individual to increased collapsibility of the upper airway are major risk factors for OSA. Given the high prevalence, obesity is the greatest risk factor for increased upper airway collapsibility and OSA. Increased adiposity results in fat deposits within pharyngeal muscles and other soft tissues surrounding the airway. OSA severity can improve with substantial weight loss, while the prevalence of OSA continues to increase as obesity escalates (Vgontzas et al., 1994; Park et al., 2011). Adenotonsillar tissue also increases airway collapsibility and the likelihood of OSA, and in children, pharyngeal lymphoid tissue is a major cause of OSA (Fregosi et al., 2003; Dayyat et al., 2009). Recently, however, obesity has become an important risk factor for OSA in children, particularly adolescents (Gozal and Kheirandish-Gozal, 2007, 2009; Bourke et al., 2011a,b). Micrognathia, retrognathia, and maxillary insufficiency also predispose to OSA. These conditions may occur as isolated conditions or as one characteristic of several craniofacial syndromes that also result in impingement on the upper airway, for example, Down’s syndrome, Treacher Collins and Pierre Robin syndrome (Kent et al., 2010). Chronic nasal obstruction from allergies or nasal polyps are additional predisposing factors for a collapsible upper airway and OSA (Dempsey et al., 2010). Supine positioning can worsen the severity of OSA in individuals, and lateral positioning for sleep may improve OSA in some individuals (Heinzer et al., 2012).

Obstructive sleep apnea can be effectively treated with the use of continuous positive airways pressure (CPAP) therapy, an approach that stents open the upper airway with positive pressure. While CPAP is highly effective in most individuals in alleviating apneas, to maintain this positive pressure in the pharyngeal lumen, a tightly fitting mask must be applied over the nose and/or mouth. Adherence with CPAP therapy is reported between 30 and 60% of patients (Weaver and Sawyer, 2010). Predictors of adherence include sleepiness, severity of OSA, claustrophobia, side effects from the therapy, patient education, and psychological well-being and support (Weaver and Sawyer, 2010). There are other options for OSA therapy including a mouthpiece that closes the jaw and advances the mandible, lateral positional therapy (as described above), and pharyngeal surgery to reduce soft tissue. In general, substantial resolution of severe OSA is unlikely with these non-CPAP therapies; thus, CPAP is recommended for all individuals with moderate to severe OSA or symptomatic OSA, regardless of severity.

Obstructive sleep apnea is a relatively recently appreciated disorder, one with phenotypes still being built. OSA has been described in medical literature for less than 50 years (Guilleminault et al., 1975). Only within the past 25 years has the disorder been considered as a potential contributor to neurobehavioral and cardiovascular abnormalities, and only recently has OSA been widely recognized as an important source of neurobehavioral and cardiovascular morbidity (Kales et al., 1985; Bhattacharjee et al., 2009; Lavie, 2009; Peppard, 2009). Intriguingly, the severity of OSA does not always predict morbidities, and there are subgroups, as yet to be defined, of patients with increased susceptibility to OSA complications, including cognitive impairments. This review serves to update and integrate the neurobehavioral, imaging, and electrophysiological phenotypes of OSA.

## Acute Physiological Disturbances in OSA

Obstructive sleep-disordered events have substantial effects on both hemodynamics and gas exchange that, in turn, influence brain health and function. As mentioned above, individuals with OSA rely upon upper airway dilator muscles surrounding the airway to stent the pharynx open. In sleep, tone in these muscles is reduced, and the pharynx is prone to collapse. When collapse occurs, larger attempts by the ventilatory pump muscles (e.g., the diaphragm) against a closed pharynx lower the intrathoracic pressure (Dempsey et al., 2010). The lower pressure reduces cardiac preload and increases cardiac afterload, thereby compromising cardiac blood flow (Scharf et al., 1992). At the same time the collapsed upper airway prevents gas exchange, thereby allowing arterial carbon dioxide to rise and oxygen to fall. The fall in oxygen is in part determined by central obesity, where the greater the abdominal obesity, the more quickly and profoundly oxygen will fall. The gas exchange interruption results in increasing sympathetic drive and arterial resistance, further challenging cardiac output. Both hypercapnia and upper airway sensory stimulation contribute to arousal and restoration of upper airway patency. Restoration of the patent airway, in turn, improves both preload and afterload, thereby suddenly increasing cardiac output and sympathetic activity (Dempsey et al., 2010). The increased cardiac output and sympathetic drive result in large increases in blood pressure. In individuals with severe OSA, systolic blood pressures may be higher in sleep than in waking. Fluctuations in carbon dioxide levels can perturb central nervous system arterial blood flow (Foster et al., 2009; Furtner et al., 2009). The frequent hypoxia/reoxygenation events impose significant oxidative stress on the brain, and increased sympathetic drive increases coagulability (both platelet and clotting factor activation) throughout the body, including the brain (Li and Veasey, 2012), while repeated arousals from obstructive breathing events result in significant sleep disruption. A recent study suggests that vibratory trauma modeling snoring vibrations can increase carotid artery endothelial damage (Cho et al., 2011). Any, or all, of these perturbances may contribute to the neurobehavioral sequelae of OSA.

## Cognitive Function in Individuals with OSA

Sleepiness, mood disturbances, and cognitive dysfunction are frequently observed in individuals with OSA. Sleepiness is the most common symptom in OSA (Engleman and Joffe, 1999). Sleepiness, measured objectively with the multiple sleep latency test and maintenance of wakefulness test or subjectively using the Epworth or Stanford sleepiness scales, is evident in most, but not all, individuals with OSA (Patel et al., 2003). Sleepiness typically improves in OSA after initiation of CPAP therapy. However, if sleepiness is severe at baseline, complete reversal of sleepiness is unlikely (Patel et al., 2003; Antic et al., 2011). The incomplete reversal with effective therapy suggests either additional causes of sleepiness or irreversible injury to neural circuits involved in sleep/wakefulness and alertness (Antic et al., 2011). To explore reasons for residual sleepiness in treated OSA, Vernet et al., compared subjects treated for OSA with and without residual sleepiness and non-OSA subjects. Participants with primary sleep disorders, insufficient sleep time, and lifelong sleepiness were excluded from the study. They found that individuals with OSA and residual sleepiness following effective therapy also had reduced stage N3 (slow wave or deep) sleep, higher depression indices, and increased periodic limb movements. While these findings are consistent with post-hypoxic injury to monoaminergic neurons, it is also possible that the sleepiness could be secondary to depressive symptomatology or idiopathic hypersomnolence (Vernet et al., 2011). Future studies are needed to follow individuals across the progression from benign snoring to OSA to determine whether OSA can result in lasting hypersomnolence.

Not all patients with OSA have cognitive abnormalities, and most of the studies finding significant differences in cognitive function have examined individuals with severe OSA. Kales et al., performed a vast array of neurobehavioral tests in subjects with and with out OSA to identify psychosocial and cognitive disturbances in severe OSA. In a study of 50 individuals with severe OSA, 76% of subjects with OSA had significant cognitive abnormalities, including disturbances in mood, thought processing, memory, communication capabilities, and attention (Kales et al., 1985). The most common mood disturbance in OSA is depression. Clinically significant depression is observed in half of all patients with moderate-severe OSA (Engleman and Douglas, 2004; Aloia et al., 2005). The high prevalence of depression in OSA has been confirmed in a more recent studies (El-Sherbini et al., 2011). That OSA therapy improves mood supports a direct relationship between OSA and depression. Decrements in vigilance and attention can also be quite profound in OSA. Attentional deficits in OSA contribute to increased risk of motor vehicle accidents. As observed with depression and sleepiness, treating OSA improves attention and reduces motor vehicle risk (Sassani et al., 2004). Impaired attention can negatively influence many, but not all, performances in neurobehavioral tests. Memory (semantic, episodic, and working domains) can be examined independent of attention. In OSA, verbal memory is impaired in all three domains; while visual memory appears unaffected by OSA (Twigg et al., 2010). Whether verbal memory improves with OSA therapy has not been determined. One caution with interpreting results of this study is that while neurological disease and diabetes were excluded, obesity was present only in the OSA group, and obesity is also a risk factor for poor cognitive function (Sellbom and Gunstad, 2012). Clearly determining how OSA and obesity influence cognitive performance will be important to discern. Subjective quality of life disturbances observed in OSA, including perceived poor social interactions, emotional instability, and reduced vitality, also improve significantly with effective therapy (Jenkinson et al., 1999). Executive function includes verbal reasoning, planning, attention, and problem solving. Deficits in each of these skills are observed in OSA, and there are important associations between the severity of hypoxemia and cognitive impairment (Carlson et al., 2011). Unlike attention, sleepiness and mood, executive function impairments in OSA are less likely to reverse with CPAP therapy (Lau et al., 2010; Saunamaki et al., 2010; Hong et al., 2012). Whether the persistent deficits represent irreversible injury from OSA or injury from another disorder or condition requires further study. It is important to recognize that most neurobehavioral studies are carried out on small sample sizes and, at the same time, have performed numerous independent behavioral tests within the same study. Thus, most OSA neurobehavioral studies are underpowered for excluding cognitive impairments. One of the most intriguing recent studies on cognitive function and OSA is the study by Canessa et al. (2011) in which subjects with severe OSA had impaired performances on most cognitive tests and several lesions by magnetic resonance imaging. With therapy, both cognitive testing and imaging abnormalities improved in parallel. Whether the imaging abnormalities represent true neuronal changes or vascular changes and why these patients showed complete normalization in cognitive function after therapy will require further study. Children with OSA may also show cognitive deficits, that cannot be explained by obesity or cardiovascular disease.

In young children without obesity, Barnes et al. (2012) identified significant visual attention deficits and learning and memory impairments in young children (4–8 years) with OSA. Impaired performance could be predicted by evoked potential testing during an attention task. Evoked potential testing during polysomnography might identify children at risk for cognitive dysfunction form OSA. This is, indeed, an area in need of careful, rigorous studies designed to identify additional causes of cognitive dysfunction in this patient population and to follow function across longer periods of therapy.

## Is Cognitive Dysfunction in OSA Consistent with Aging?

The above clinical studies provide strong evidence that OSA contributes to brain dysfunction and that treating OSA improves neurobehavioral outcomes. Advanced aging is a ubiquitous insult to brain function, and many of the cognitive abnormalities (e.g., verbal memory, reaction time, and executive function) observed in OSA are also seen with aging. Whether OSA represents a distinct singular event brain injury irrelevant of aging, a continuous injury with no influence on aging, or whether OSA injury directly influences aging injury will be important to discern. A few age-OSA studies have been performed with negative results. Two small cross-sectional studies found no effect of OSA on age-related cognitive function (Mathieu et al., 2008; Sforza et al., 2010). Alchanatis et al. (2004) have shown that middle-aged individuals with OSA have more cognitive impairment than age-matched controls, while younger individuals with OSA appear similar to controls. Younger individuals with OSA show brain activation (by functional magnetic resonance imaging) in verbal and attention cognitive tasks and do not have impaired performance. In contrast, middle-aged individuals with OSA do not show brain activation and have impaired performances, suggesting that OSA and aging are additive (Ayalon et al., 2010). Alternatively a longer duration of OSA may worsen compensatory mechanisms. Future prospective studies will be helpful to elucidate the relationship between aging and OSA effects on cognition.

Newer study designs implemented for aging and cognitive function studies use within subject longitudinal analysis and are, therefore, far more sensitive at detecting differences. For example, older cross-sectional studies identified the beginning of decline in cognition with aging to be around 60 years of age. Recent longitudinal studies using within subject analysis across time offer discouraging news: the decline begins much earlier. In the large Whitehall series in Great Britain (over 7000 subjects), adults ranging in age from 45 to 70 years at the start of the study were followed with cognitive tests for 10 years to allow within subject comparisons with time. Executive functions including reasoning, memory, and verbal fluency, declined in all age groups including 45–49-year-olds. In contrast, vocabulary was largely unaffected across the 10 years for any age groups. Declines for males and females were similar, and the amount of decline across 10 years was similar for all tests, except reasoning, which plummeted more dramatically in the older groups. Recently a sub-analysis of the data found that changes in total sleep times between tests were associated with poorer cognition. While the executive function impairments in OSA are consistent with those observed across aging, sleepiness, and reduced vigilance observed with OSA are not observed in healthy aging at least not before age 80. The marked sleepiness and impaired vigilance suggest a distinct process unrelated to aging. To carefully examine interactions between aging and OSA, longitudinal examinations of verbal executive function in individuals with and without OSA are needed.

## Brain Imaging in OSA

Imaging studies of the brains of individuals with and without OSA have revealed distinct regional metabolic disturbances and gray and white matter abnormalities. Proton magnetic resonance spectroscopy (PMRS) provides a window into brain metabolism. *N*-acetylaspartate (NAA) is a marker of active neurons, and choline (cho) indicates cell membranes, while creatinine (cr) marks energy metabolism. Thus, a low NAA/cho or low NAA/cr indicate reduced viable neurons within the region measured. Kamba et al. (1997) examined 23 subjects (aged 24–75 years) and 15 controls finding significantly reduced NAA/cho in the cerebral white matter in individuals with moderate-severe OSA, while individuals with mild OSA showed no differences. The researchers performed a follow-up study excluding individuals with diabetes and hypertension that might confound results and found that the severity of OSA predicted cerebral white matter reductions in NAA/cho (Kamba et al., 2001). Two other small studies examining NAA/cr in OSA and control subjects found reduced NAA/cr in the hippocampus (Bartlett et al., 2004) and throughout the cerebral white matter (Alchanatis et al., 2004). A more recent study by Tonon et al. (2007) found reduced NAA in the cerebral cortex in OSA subjects with sleepiness but without other cognitive abnormalities. This work supports the concept that PMRS could be used in early detection of brain injury before cognitive impairments. Importantly, in this same study, patients were then treated with CPAP for 6 months and re-examined (Tonon et al., 2007). The reduced cortical NAA did not reverse. Another way to characterize brain metabolics is with ^31^P PMRS, measuring ATP and metabolites. In severe untreated OSA subjects with >10% sleep time spent with hypoxemia, ATP levels (measured for the whole forebrain) are reduced during sleep (Tonon et al., 2007). One of the most striking sets of findings involves NAA measures in children with OSA. Halbower et al. (2006) examined 33 children (6–16 years) with OSA and 12 controls. In children with OSA, as previously shown in adults, NAA was reduced in the right hippocampus and frontal cortex. These reductions were associated with reduced intelligence quotients (IQ’s) and school performance (Halbower et al., 2006). There are two small reports suggesting a negative relationship between obesity and NAA/cr (Gazdzinski et al., 2008, 2010). In these studies OSA was not excluded, and it is possible that OSA contributes to the reduced NAA in obesity. Future studies are necessary to discern whether obesity contributes directly to the reduced NAA in OSA.

A number of studies have compared OSA and non-OSA white and gray matter volumes using magnetic resonance imaging (MRI). Macey et al. (2002) examined male subjects (aged 25–70) with moderate-severe OSA and normal healthy controls and found that while gray matter declined across ages studied in controls, values were reduced in young OSA subjects and were not lower with age. These results are more consistent with a single hit from OSA on brain morphology rather than accelerated aging changes. In OSA, gray matter lesions were identified in the frontal and parietal cortex, the right hippocampus, and deep cerebellar nuclei (Macey et al., 2002). Morrell et al. (2003) confirmed hippocampal gray matter loss in severe OSA; although her group did not confirm cortical gray matter losses. Thomas et al. (2005) examined functional MRI responses to a verbal memory task in 16 very severe OSA subjects (21–50 years) and 16 age-matched controls, finding impaired verbal memory with reduced prefrontal cortex activation responses in OSA subjects. After 2 months+ CPAP therapy, repeat imaging showed minimal improvement, with no improvements in prefrontal cortex inactivation. Others have also observed reduced frontal activation with memory tasks in OSA (Ayalon et al., 2009a,b; Zhang et al., 2011), although patients with less severe OSA may have compensatory (increased activation) responses (Ayalon et al., 2009b). It is important to acknowledge that there is one MRI study with negative gray matter findings (O’Donoghue et al., 2005). O’Donoghue et al., examined 27 subjects with OSA and 24 controls. As a group, the OSA subjects had a mean Epworth sleepiness score of 13, demonstrating group sleepiness. No regional reductions in gray matter were identified (O’Donoghue et al., 2005). A follow-up study by the same research group with larger sample sizes (adequately powered to detect differences) found gray matter lesions in the temporal gyrus (frontal cortex) and cerebellum (Morrell et al., 2010). Using a newer, more sensitive methodology, diffusion tensor imaging with fractional anisotropy, Macey et al. (2008) identified substantial white matter lesions in OSA, largely throughout the brain but with more pronounced changes in the cortices, limbic system, pons, and cerebellum tracts. Perhaps the most intriguing study is the recent study by Canessa et al., in which severe OSA subjects with sleepiness, depression, and neurobehavioral abnormalities had, as expected from other studies, reduced gray matter volumes in the hippocampus and fronto-parietal cortices. The surprising finding was that with CPAP therapy, cognitive performance normalized, as did gray matter lesions. In fact, cognition and morphology changed in parallel with therapy. While these findings require replication to determine whether there was something unique about this population, the results demonstrate the importance of effective therapy for OSA.

In summary, there are significant reductions in neuron metabolite measures suggesting injured, non-viable neurons in particular brain regions, including the frontal cortex and hippocampus. Of concern, these changes are also observed in children, and in some children and adults the abnormalities are not reversed with therapy. It will be important to discern obesity changes from OSA changes with future studies. In addition there is significant gray matter loss in OSA, with the hippocampus being most sensitive to gray matter loss in OSA. There is one study suggesting improvement in gray matter lesions with CPAP therapy. Newer fractional anisotropy MRI studies find substantive white matter injury in OSA. While expensive and labor intensive, brain-imaging studies provide valuable temporal windows into brain morphology of OSA.

There are some interesting similarities between imaging findings in OSA and aging in that the brain regions affected in OSA are similar to brain regions most vulnerable in aging. Specifically, the frontal cortex (central to executive function) white and gray matter decline with aging, as does hippocampal volume (Michielse et al., 2010; Schulze et al., 2011; Taki et al., 2011a,b). Newer longitudinal studies show healthy age-related changes in both white matter tracts (using fractional anisotropy); reduced hippocampal activation upon relevant task (functional MRI), and measurable cortical gray matter changes over, incredibly, a 1–2 year period (Fjell et al., 2009; Nyberg et al., 2010; O’Brien et al., 2010; Teipel et al., 2010; Cardenas et al., 2011). Annual decline in cortical volume in healthy individuals over age 60 years is 0.5%, with steeper declines in older individuals (Fjell et al., 2009; Nyberg et al., 2010). These same regions are susceptible to larger volume losses in individuals with mild cognitive impairment or Alzheimer’s disease. Involvement of the same regions does not implicate the same pathogenesis in OSA, aging, and Alzheimer’s, but rather highlights the neural tissue most vulnerable to diverse insults. A next important step in OSA is to determine if the changes across time in individuals with OSA are closer to those expected for healthy aging or those observed in Alzheimer’s and mild cognitive impairment. Whether treatment of OSA lessens neural volume loss across time, in parallel with less behavioral decline would be extremely important to discern as well.

## Nerve Conduction Defects in OSA

Peripheral nerve conduction studies might provide an important functional window into OSA neural injury. However, there are only a few studies reporting peripheral nerve conduction characteristics in individuals with OSA. One of the first studies was a case series study. In seven individuals diagnosed with severe OSA, four had phrenic nerve conduction delays (Lu et al., 1998). Mayer et al. (1999) found markedly reduced median nerve amplitudes and reduced conduction velocity in individuals with severe OSA. Peripheral neuropathy may also contribute to erectile dysfunction (increased prevalence with OSA). Men with severe OSA have impaired bulbocavernosus reflex responses with prolonged nerve conduction latencies and reduced amplitude (Fanfulla et al., 2000). Most compelling is a recent study by Dziewas et al. (2007) in which sural nerve amplitudes were measured in individuals with and without OSA (wide range of ages/severity). Sensory nerve amplitudes were reduced by 45%. Less than half of the subjects used CPAP after the initial studies. In these individuals, sural nerve amplitude abnormalities partially reversed, while in non-treated OSA subjects no change was observed (Dziewas et al., 2007). These data strongly support a direct injury to peripheral nerves in OSA. Clearly large-scale studies are needed in children and adults to determine the prevalence and reversibility of peripheral neuropathy in OSA. Future studies are needed to relate peripheral nerve findings with cognitive function and brain imaging in OSA. If cognitive performance declines in OSA in parallel with peripheral nerve conductance abnormalities, the latter would provide a simple relatively inexpensive screening tool for OSA neural injury. Ultimately blood or urine biomarkers for OSA phenotypes should be an important direction.

## Cerebrovascular Disease in OSA

Clinical studies demonstrate strong associations between cerebrovascular disease and OSA, and this, too, may contribute to cognitive dysfunction in OSA. In a prospective study of over 5,000 subjects followed for almost 9 years, moderate-severe OSA is an independent risk factor for cerebrovascular disease in men (twofold increase in risk), yet in women, OSA does not appear to be a significant independent risk factor for cerebrovascular disease (Redline et al., 2010). Patients hospitalized with acute onset stroke and who have OSA have improved outcomes (less impairment) when treated with CPAP during rehabilitation hospitalization (Ryan et al., 2011). The mechanisms underlying cerebrovascular disease in untreated OSA are not established, but several recent studies highlight potential sources of cerebrovascular injury in OSA. First, there are impaired autonomic responses including cerebrovascular responses to hypoxia in individuals with OSA (Pizza et al., 2010). Following an apnea there is a large drop in cerebral blood flow velocity that could be rather detrimental following hypoxia (Balfors and Franklin, 1994; Hajak et al., 1996). The impaired responses promote large fluctuations in cerebral hemodynamics. Cerebrovascular responses are further impaired by reduced carbon dioxide vasodilatation (Morgan et al., 2010). One of the most interesting risks for cerebrovascular disease is that snorers have increased carotid atherosclerosis (Lee et al., 2008). A direct link to vibratory trauma was recently demonstrated in a rabbit model where vibratory damage resulted in endothelial damage, an important precursor to atherosclerosis (Cho et al., 2011). Future studies examining the risk of snoring for atherosclerosis in distant arteries and longitudinal studies in snorers will help identify the relationship between snoring and atherosclerosis.

## Neurobehavioral Manifestations in Animal Models of OSA

There are distinct advantages and disadvantages with studying animal models of a particular disease. If a study animal is otherwise healthy, then an isolated anomaly can be tested for a direct link with morbidities. Frequently, however, animal models are incomplete models of the disease and can provide supportive rather than definitive information. The English bulldog is one of the few natural animal models of OSA (Hendricks et al., 1987); although most of the events are observed in REM sleep. The English bulldog has several relatively commonly occurring genetic anomalies, including palate and spine defects and hydrocephalus, which may influence OSA and/or cognitive function. Most other non-human species have well-developed hyoid arches to protect the upper airway and are less likely to have obesity. Leptin deficient rodents are obese and have increased upper airways resistance but do not evidence apneas (Ray et al., 2007). Several rodent models have been developed to examine the neural effects of long-term intermittent hypoxia (LTIH), intermittent hypercapnia, and sleep fragmentation of cognitive function. Rodent models have the advantages of: ready availability, low-cost, and the availability of genetic models that may be used to identify mechanisms of brain injury. LTIH, modeling severe OSA oxygenation patterns (40 events/h with oxygen saturation nadirs of 75%), in mice results in irreversible sleepiness with injury to and loss of catecholaminergic wake neurons (Zhu et al., 2007). Whether more mild exposures cause injury or wake impairments has not been explored. Neuronal death is observed in neonatal rats exposed to both intermittent hypercapnia and LTIH (Douglas et al., 2010), but whether the neuronal loss contributes to cognitive impairments will require further study. Exposure to severe OSA oxygen patterns in young adult rats has been shown to impair spatial learning and memory, with injury to a behaviorally relevant brain region, the hippocampus (Gozal et al., 2001). Sleep fragmentation, modeling OSA sleep disruption, induces comparable spatial learning and memory impairments (Nair et al., 2011), and LTIH impairs attentional set shifting (the ability to focus on important stimuli) in rats (McCoy et al., 2010). A more comprehensive OSA model (one with sleep state-dependent obstructive apneic events) was developed recently (Neuzeret et al., 2011). This model has intermittent hypoxemia, intermittent hypercapnia, hemodynamic changes as in OSA, and sleep fragmentation, making this a much more comprehensive model of OSA in which to study neural injury. It will be of great interest to determine whether the composite of physiological disturbances in this model results in different or more pronounced neurobehavioral impairments. Collectively, the present data strongly support the concept that OSA physiological perturbances, including LTIH, hypercapnia, and sleep fragmentation directly impair brain function and injure neurons, contributing to impaired attention, wakefulness, and spatial memory.

Consistent with hippocampal lesions identified in OSA, LTIH results in hippocampal-dependent learning and memory impairments in young adult rats and mice (Gozal et al., 2001). LTIH exposure is associated with behavioral impairments, oxidative, inflammatory responses, and apoptosis changes have been identified in the CA1 region of the hippocampus in animals exposed to LTIH (Li et al., 2003, 2004). Some of the molecular changes are evident only in early injury. For example, inducible nitric oxide synthase protein is elevated only in the first week of LTIH (Li et al., 2004). There are several proteins that influence the vulnerability to LTIH spatial learning and memory impairments. Transgenic reduction in platelet activating factor improves spatial learning and memory performances after LTIH (Reeves and Gozal, 2004). Administration of catechin polyphenols or growth hormone across LTIH exposure offers some protection (Burckhardt et al., 2008). Allowing physical activity of rodents during LTIH prevents spatial learning and memory impairments (Gozal et al., 2010). It is both encouraging and discouraging that there are so many modifiers and seemingly so many pathways are involved in LTIH cognitive impairment. On the optimistic side there are many ways to protect the brain from injury. Regular exercise, green tea (for catechin polyphenols) and adequate sleep time (to boost growth hormone) may help confer resistance to LTIH and possibly OSA neural injury. From the pessimistic perspective, perhaps we have not found the mechanisms underlying OSA and LTIH injury; we are simply improving general brain health. To reconcile this concern requires testing both the presence of LTIH modified pathway and the complete effects of both increasing and reducing the molecule under examination.

Apneas may increase neural injury to a greater extent than intermittent hypoxia. The brain, over other organs, may be uniquely sensitive to recurrent apneas because of greater increases in oxygen tension across time (Almendros et al., 2011). Almendros et al. (2011) developed parallel rat models of intermittent hypoxia and recurrent apnea and then measured tissue oxygen tension across repeated events. Across time, muscle and visceral fat oxygen tensions with repeated events did not change. In contrast, in the cerebral cortex, there was a progressive increase in oxygen tension values for both the peak and nadir of apneic events. This suggests that vasoactive changes in response to apneas, but not intermittent hypoxia influence the oxygen tension in the cortex. The cortex of the brain but not muscle or fat in the apnea model showed reduced glutathione supporting oxidative stress (Almendros et al., 2011).

One of the molecules that likely contributes directly to LTIH injury is nicotinamide adenine dinucleotide phosphate (NADPH) oxidase. We have identified that LTIH in mice results in irreversible wake impairments (Veasey et al., 2004). LTIH-exposed mice sleep more during their active (subjective day) and inactive (subjective night) periods. In a mouse version of the multiple sleep latency test, mice exposed to LTIH fall asleep much earlier. LTIH induces oxidative and nitrative stress in regions of wake active neurons, manifested as increased tyrosine nitration, 8-, 12-isoprostanes, and carbonyl protein levels (Zhan et al., 2005a,b). NADPH oxidase subunits are up-regulated and activated in wake regions in response to LTIH (Zhan et al., 2005b). Moreover, mice with NADPH oxidase inhibited across LTIH or mice with transgenic absence of the NADPH oxidase isoform that is up-regulated in response to LTIH (NADPH oxidase 2) the mice do not develop wake neuron injury or sleepiness. Moreover, mice with absent or inhibited NADPH oxidase 2, developed less inflammatory response to LTIH in the brains and less nitration, oxidation (Zhan et al., 2005b; Zhu et al., 2007). Thus, it is very likely that NADPH oxidase contributes to the injury.

More recent studies have identified LTIH-induced up-regulation of hypoxia inducible factor-1a (HIF-1a) and mitochondrial and endoplasmic reticulum injury (Douglas et al., 2010; Prabhakar et al., 2010). NADPH oxidase and HIF-1a have bidirectional positive feedback on each other in LTIH, so that inhibiting or reducing one lowers the LTIH response of the other and in doing so lessens injury. The next steps are to confirm in post-mortem OSA brain tissue that NADPH oxidase is activated and to determine how NADPH oxidase is activated in the brain in LTIH and OSA. Hypoxia can activate angiotensin 1A and result in NADPH oxidase activation, and angiotensin 1A antagonists can reduce NADPH oxidase activation. It will be of interest if angiotensin 1A antagonists confer protection across time in cognitive and brain morphology changes in individuals with OSA.

## Conclusion

Collectively from studies performed over the past two decades, a constellation of specific cognitive impairments, brain morphology, and electrophysiological findings in OSA has developed. OSA is not only associated with hypersomnolence, depression, poor attention, impaired verbal memory, poor reasoning, and planning, but therapy for OSA can improve each of these findings. Presently, some of the larger questions left unanswered include: which patients with OSA are at risk for neural injury, and can we prevent progression of neuronal injury? Which neural injuries in OSA are irreversible? How do we identify those individuals at risk for brain injury and dysfunction from OSA? Are there markers, such as evoked potentials or electromyography that can predict central injury and dysfunction? By piecing together this more comprehensive phenotype (imaging, electrophysiology, and behavioral testing) in future longitudinal studies with patient characteristics, it is likely that many of these questions can be answered.

## Conflict of Interest Statement

The author declares that the research was conducted in the absence of any commercial or financial relationships that could be construed as a potential conflict of interest.
